# Role of the PGE_2_ receptor subtypes EP1, EP2, and EP3 in repetitive traumatic brain injury

**DOI:** 10.1111/cns.13228

**Published:** 2019-10-16

**Authors:** James Catlin, Jenna L. Leclerc, Krunal Shukla, Sarah M. Marini, Sylvain Doré

**Affiliations:** ^1^ Department of Anesthesiology University of Florida Gainesville FL USA; ^2^ Department of Neurology, Psychiatry, and Pharmaceutics University of Florida Gainesville FL USA; ^3^ Present address: Department of Anesthesiology and Perioperative Medicine Oregon Health & Science University Portland OR USA

**Keywords:** concussion, eicosanoids, EP receptors, prostanoids, repetitive head injury

## Abstract

**Aims:**

The goal was to explore the signaling pathways of PGE_2_ to investigate therapeutic effects against secondary injuries following TBI.

**Methods:**

Young (4.9 ± 1.0 months) and aged (20.4 ± 1.4 months) male wild type (WT) C57BL/6 and PGE_2_ EP1, 2, and 3 receptor knockout mice were selected to either receive sham or repetitive concussive head injury. Immunohistochemistry protocols with Iba1 and GFAP were performed to evaluate microgliosis and astrogliosis in the hippocampus, two critical components of neuroinflammation. Passive avoidance test measured memory function associated with the hippocampus.

**Results:**

No differences in hippocampal microgliosis were found when aged EP2^−/−^ and EP3^−/−^ mice were compared with aged WT mice. However, the aged EP1^−/−^ mice had 69.2 ± 7.5% less hippocampal microgliosis in the contralateral hemisphere compared with WT aged mice. Compared with aged EP2^−/−^ and EP3^−/−^, EP1^−/−^ aged mice had 78.9 ± 5.1% and 74.7 ± 6.2% less hippocampal microgliosis in the contralateral hemisphere. Within the EP1^−/−^ mice, aged mice had 90.7 ± 2.7% and 81.1 ± 5.6% less hippocampal microgliosis compared with EP1^−/−^ young mice in the contralateral and ipsilateral hemispheres, respectively. No differences were noted in all groups for astrogliosis. There was a significant difference in latency time within EP1^−/−^, EP2^−/−^, and EP3^−/−^ on day 1 and day 2 in aged and young mice.

**Conclusion:**

These findings demonstrate that the PGE_2_ EP receptors may be potential therapeutic targets to treat repetitive concussions and other acute brain injuries.

## 1 INTRODUCTION

Traumatic brain injury (TBI) is a major public health concern that is characterized as a structural and physiological injury, which leads to neurological damage and dysfunction.^1^, ^2^, ^3^, ^4^ In 2013, the Centers for Disease Control and Prevention (CDC) identified 2.8 million cases of TBI and 56 000 TBI‐related deaths.^5^ TBI has damaging and enduring effects, which are typically characterized by changes in emotion, executive function, language, and disposition.^6^ Additionally, TBI has acute sequelae that increase morbidity and mortality following the traumatic event, including acute respiratory failure, pneumonia, and various infections, as well as debilitating chronic sequelae, such as sleep disorders, anxiety, depression, and posttraumatic stress disorder.^6^

TBI results in both primary and secondary damage.^1^ Primary damage after TBI is the direct consequence of the physical trauma, specifically the distortion of the brain tissue that often results in disturbance of normal brain function.^1^ Secondary damage after TBI is indirect, such as the neuroinflammatory response that follows primary injury.^1^, ^7^ Unfortunately, not much can be done clinically to reverse the primary damage of TBI given the mechanism of injury.^1^ Clinical treatment of TBI, therefore, focuses on the prevention of secondary damage that arises after the primary trauma.^1^ Since neurological inflammation is partly mediated through increased secretion of the lipid metabolite prostaglandin E_2_ (PGE_2_), this paper explores the signaling pathways of such eicosanoids to discover potential biological targets to clinically mitigate secondary brain damage.^7^

PGE_2_ is synthesized from arachidonic acid, a polyunsaturated omega‐6 fatty acid, through the cyclooxygenase‐2 (COX‐2) pathway.^7^, ^8^ It is highly implicated in the initiation of inflammatory processes, specifically increasing vascular permeability, fever, and hyperalgesia.^8^ Furthermore, fever and vasogenic edema (as a result of increased vascular permeability) are common acute sequelae after TBI and have been suggested as independent poor outcome predictors.^9^ The rise in biological PGE_2_ after a neuroinflammatory incident has both neurotoxic and neuroprotective effects.^7^ The exact effect depends on which E_2_ prostanoid (EP) receptor subtype that PGE_2_ activates and the underlying neuropathological process.^7^, ^10^

The four main E_2_ prostanoid (EP) receptor subtypes, correspondingly named EP1, EP2, EP3, and EP4, are G protein‐coupled receptors that interact with PGE_2_ and activate their own distinctive signaling cascade pathways.^7^, ^11^ PGE_2_ binding to the EP1 receptor results in an increase in intracellular Ca^2+^ levels.^11^ The exact mechanism in which Ca^2+^ increases, however, is still being investigated.^11^ The EP2 receptor and the EP4 receptor, following PGE_2_ binding, activate adenylate cyclase, leading to an increase in cAMP, which binds to the regulatory subunits of protein kinase A (PKA) to release its catalytic subunits that will phosphorylate various cellular targets.^11^ Various human EP3 receptor isoforms have been identified. Following PGE_2_ binding, certain EP3 receptor isoforms that are G_i_‐mediated inhibit adenylate cyclase and increase intracellular Ca^2+^ levels.^11^ Other EP3 receptor isoforms that are also G_i_‐mediated activate the MAPK pathway upon PGE_2_ binding, resulting in transcriptional activation.^11^

EP receptors can modulate various outcomes depending on the injury model under investigation. For example, EP2 receptor signaling has specifically demonstrated both neuroprotective and neurotoxic effects depending on the underlying neuropathological pathway.^7^, ^10^ The neuroprotective effects of EP2 signaling have been demonstrated following hypoxic/ischemic/excitotoxic injuries.^12^, ^13^ Oxidative stress injuries and pathologies have, on the other hand, displayed neurotoxic effects through EP2 signaling.^1^, ^7^ Secondary damage after TBI involves hypoxia and oxidative stress, which modify the balance of neuroinflammatory pathways.^7^

Neuroinflammation involves astrogliosis, which has both beneficial and deleterious effects depending on the astrocyte concentration.^14^ Astrogliosis may mitigate the spread of damage and stifle neuronal repair mechanisms through the inhibition of axonal regrowth.^14^ Astrogliosis is, therefore, necessary for healing, but in excess, it may induce neurotoxicity.^14^ Neuroinflammation is highly associated with microgliosis, which may lead to neuronal damage and has been associated with many neurodegenerative diseases.^15^

Many studies have confirmed that multiple injuries in a brief time span result in worse behavioral outcomes and histopathology in comparison to a single mild TBI. Repetitive closed‐head injuries (CHI) result in increased microgliosis, astrogliosis, and neuronal death in several brain regions, including the hippocampus and cerebellum, compared with single CHI.^16^, ^17^, ^18^ This study aims to investigate the neuroinflammatory effects of EP receptor signaling in a preclinical model of mild repetitive CHI by measuring astrogliosis and microgliosis in EP1, 2, and 3 knockout mice. The ultimate goal was to determine whether any of these EP receptor subtypes may be potential targets for the treatment of secondary damage associated with TBI.

## 2 METHODS

### 2.1 Mice

Studies were performed on young (4.9 ± 1.0 months) and aged (20.4 ± 1.4 months) male wild type (WT) (n = 8, n = 7), EP1^−/−^ (n = 10, n = 8), EP2^−/−^ (n = 8, n = 9), and EP3^−/−^ mice (n = 8, n = 11). The EP1^−/−^, EP2^−/−^, and EP3^−/−^ C57BL/6 mice developed normally, gained weight at a rate equal to that of WT mice and had no gross anatomical or behavioral abnormalities. Genotypes were established by polymerase chain reaction before experimental procedures. Colonies were housed in a temperature‐controlled environment (23.2 ± 2.0ºC) on a 12 hours‐reversed dark/light cycle. Mice had sufficient access to food and water before and after surgical procedures. The Institutional Animal Care and Use Committee at the University of Florida approved all animal protocols.

### 2.2 Repetitive concussive head injury (rCHI)

Mice were selected to either receive sham or rCHI, consisting of four total impacts separated by 24‐hour intervals. This experimental strategy was determined based on previous studies, which indicated that the vulnerability interval for repeated mild TBI in mouse models is between 24 and 48 hours.^17^, ^19^ On the first day of surgery, mice were anesthetized using 4% isoflurane for 2‐3 minutes until the animal reached deep anesthesia. Mice were shaved, cleaned, and placed on a stereotactic frame, where a small elastic tube was used to hold the chin of the animal and a flexible nose tube was fastened to the snout, allowing for maintenance with 2%‐3% isoflurane during surgery. This method was used in place of ear bars, to avoid damaging the ear canal since impact would move the head in a downward position. The impact was controlled with a PCI3000 PinPoint Precision Cortical Impactor (Hatteras Instruments), and the center of impact was positioned to the right of the midline, midway between the coronal and sagittal sutures. A silicone tip (5 mm diameter), impact speed of 3 m/s, concussion depth of 5 mm, and a dwell time of 100 ms were used. The midline incision was closed using surgical clips, and all animals were monitored in a temperature‐ and humidity‐controlled incubator until they recovered. The same protocol was used for sham animals, except for the impact, in order to control for all other experimental variables.

### 2.3 Behavioral assessment

An apparatus with light and dark chambers connected through a sliding door was used for the passive avoidance test. When the sliding door was opened, the mice were delivered with an electrical foot shock of 0.5 mV through the metal grid on the floor in the dark chamber. Then, the mice were allowed to return to the light chamber after 30 seconds. The time that it took the mice to enter the dark chamber (maximum of 300 seconds) was recorded. After 24 hours, no electrical stimuli were used in the dark chamber, and the step‐through latency time was measured again by placing the mice into the light compartment.

### 2.4 Immunohistochemistry

Immunohistochemistry was performed to evaluate microgliosis and astrogliosis using ionized calcium‐binding adapter protein (Iba1) and glial fibrillary acidic protein (GFAP) as primary antibodies. A secondary biotinylated antibody was used for detection for both stainings. The Vectastain Elite Avidin/biotinylated enzyme complex and 3,3′‐diaminobenzidine (DAB) kits (Vector Laboratories) were used following the manufacturer's instructions for the avidin‐peroxidase step and final DAB reaction, respectively. After dehydrating the slides in increasing concentrations of ethanol, they were coverslipped with Permount. All Iba1 and GFAP slides were scanned using a ScanScope CS and were subsequently analyzed using ImageScope software (Aperio Technologies, Inc). The hippocampal regions of the mice were assessed for astrogliosis and microgliosis by a person blinded to the genotypes. Both microgliosis and astrogliosis were analyzed by outlining the hippocampal regions with 1000 by 1000 pixel boxes in the ipsilateral and contralateral hemispheres. After outlining the impacted brain regions, an appropriate ImageScope Positive Pixel Count algorithm was used for the quantification of immunohistochemical stains. Algorithms were tuned to automatically detect and calculate the appropriate signal and its strength.

### 2.5 Statistical methods

JMP (SAS) and GraphPad Prism 6 (GraphPad) software were used for statistical analysis. Data were analyzed with two‐way analysis of variance (ANOVA) followed by Tukey's test and expressed as mean ± SEM with *P* < .05 considered statistically significant.

## 3 RESULTS

### 3.1 Effect of EP1, 2, 3 receptor deletion on microgliosis in young and aged mice after rCHI

**Figure 1:**
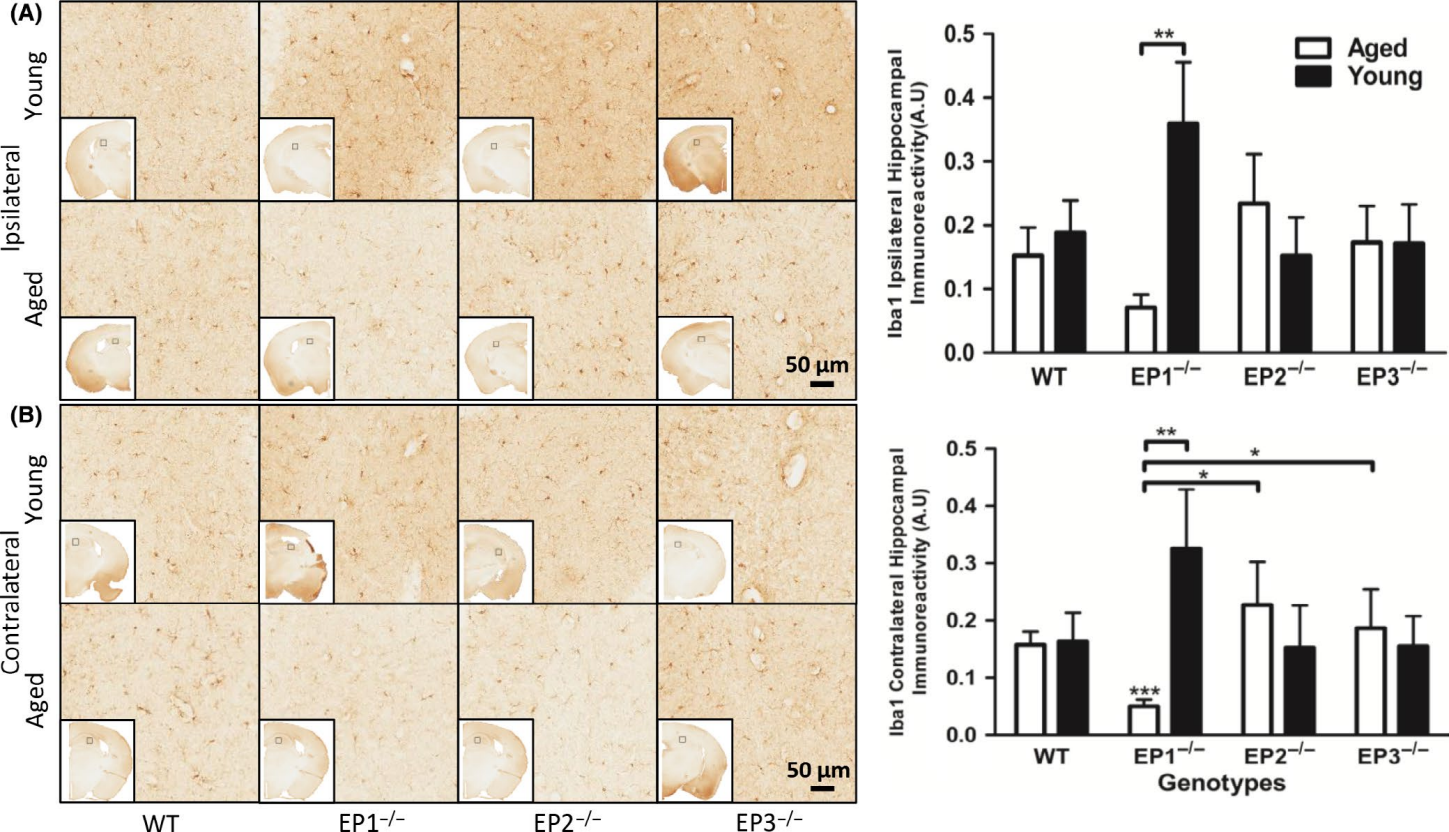
Effect of PGE_2_ EP1, EP2, and EP3 receptor deletion on microglial activation after rCHI. Seventy‐two hours after TBI, mice were sacrificed and brain sections were processed for Iba1 immunochemistry to evaluate hippocampal microgliosis and morphological changes. (A and B) Representative high magnification images of hippocampal brain sections showing the aged and young (A) ipsilateral and (B) contralateral hemispheres of WT, EP1^−/−^, EP2^−/−^, and EP3^−/−^ mice. Square selections denote the magnified regions' locations. Quantification of brown positive pixel count indicated that EP1^−/−^ aged mice had significantly less (A) ipsilateral and (B) contralateral Iba1 immunoreactivity compared with WT aged mice. (B) EP1^−/−^ aged mice had significantly less Iba1 immunoreactivity compared with EP1^−/−^ young mice. EP1^−/−^ aged mice had significantly less Iba1 immunoreactivity compared with EP2^−/−^ and EP3^−/−^ aged mice. The reduction in microglial activation was accompanied by distinct morphological changes. Comparisons included aged male and young male wild type (n = 8, n = 7), EP1^−/−^ (n = 10, n = 8), EP2^−/−^ (n = 8, n = 9), EP3^−/−^ (n = 8, n = 11), *P < .05, **P < .01

In the ipsilateral hemisphere, no significant differences in hippocampal microgliosis were found when EP1^−/−^, EP2^−/−^, and EP3^−/−^ aged mice were compared with WT aged mice (0.135 ± 0.041 vs 0.062 ± 0.018 AU, *P* *=* .077, vs 0.210 ± 0.071 AU, *P* = .882, vs 0.155 ± 0.053, *P* = .906; Figure 1A). When compared with WT aged controls, EP1^−/−^ aged mice had less hippocampal microgliosis after rCHI in the contralateral hemisphere. EP1^−/−^ aged mice had 69.2 ± 7.5% less hippocampal microgliosis in the contralateral hemisphere compared with WT aged controls (0.139 ± 0.021 vs 0.043 ± 0.010 AU, *P* *=* .0005; Figure 1B). EP2^−/−^ aged mice and EP3^−/−^ aged mice showed no significant difference in hippocampal microgliosis in the contralateral hemisphere compared with WT aged controls (0.139 ± 0.021 vs 0.204 ± 0.068 AU, *P* *=* .689 and vs 0.169 ± 0.063 AU, *P* *=* .707, respectively).

In the ipsilateral hemisphere, the EP1^−/−^ aged mice had 81.1 ± 5.6% less hippocampal microgliosis compared with EP1^−/−^ young mice (0.062 ± 0.018 vs 0.329 ± 0.091 AU, *P* *=* .002; Figure 1A). Similarly, within the EP1^−/−^ mice, aged mice had 88.7 ± 2.7% less hippocampal microgliosis compared with young mice in the contralateral hemisphere (0.043 ± 0.010 vs 0.297 ± 0.099 AU, *P* *=* .0004; Figure 1B).

Compared with EP2^−/−^ aged mice, EP1^−/−^ aged mice had 78.9 ± 5.1% less hippocampal microgliosis in the contralateral hemisphere (0.042 ± 0.010 vs 0.204 ± 0.068 AU, *P* *=* .025; Figure 1B). EP1^−/−^ aged mice had 74.7 ± 6.2% less hippocampal microgliosis in the contralateral hemisphere compared with EP3^−/−^ aged mice (0.042 ± 0.010 vs 0.169 ± 0.063 AU, *P* *=* .017). No significant difference was found in hippocampal microgliosis between the EP2^−/−^ aged mice and EP3^−/−^ aged mice in the contralateral hemisphere (0.204 ± 0.068 vs 0.169 ± 0.063 AU, *P* *=* .905).

After summation of hippocampal microgliosis positive pixel in the ipsilateral and contralateral hemispheres, EP1^−/−^ aged mice had 61.7 ± 10.3% less hippocampal microgliosis compared with the WT aged controls (0.104 ± 0.028 vs 0.274 ± 0.060 AU, *P* *=* .032). Between genotypes, EP1^−/−^ aged mice had 74.9 ± 6.8% less hippocampal microgliosis compared with EP2^−/−^ aged mice (0.104 ± 0.028 vs 0.414 ± 0.139 AU, *P* *=* .046). EP1^−/−^ aged mice had 67.6 ± 8.7% less hippocampal microgliosis compared with EP3^−/−^ aged mice (0.104 ± 0.028 vs 0.324 ± 0.116 AU, *P* *=* .028). No significant difference was found in the hippocampal microgliosis between EP2^−/−^ and EP3^−/−^ aged mice (0.414 ± 0.139 vs 0.324 ± 0.116 AU, *P* *=* .874). Within the EP1^−/−^ mice, aged mice had 83.3 ± 4.5% less hippocampal microgliosis compared with young mice (0.104 ± 0.028 vs 0.626 ± 0.188 AU, *P* *=* .0009).

### 3.2 Effect of EP1, 2, 3 receptor deletion on astrogliosis in young and aged mice after rCHI

**Figure 2:**
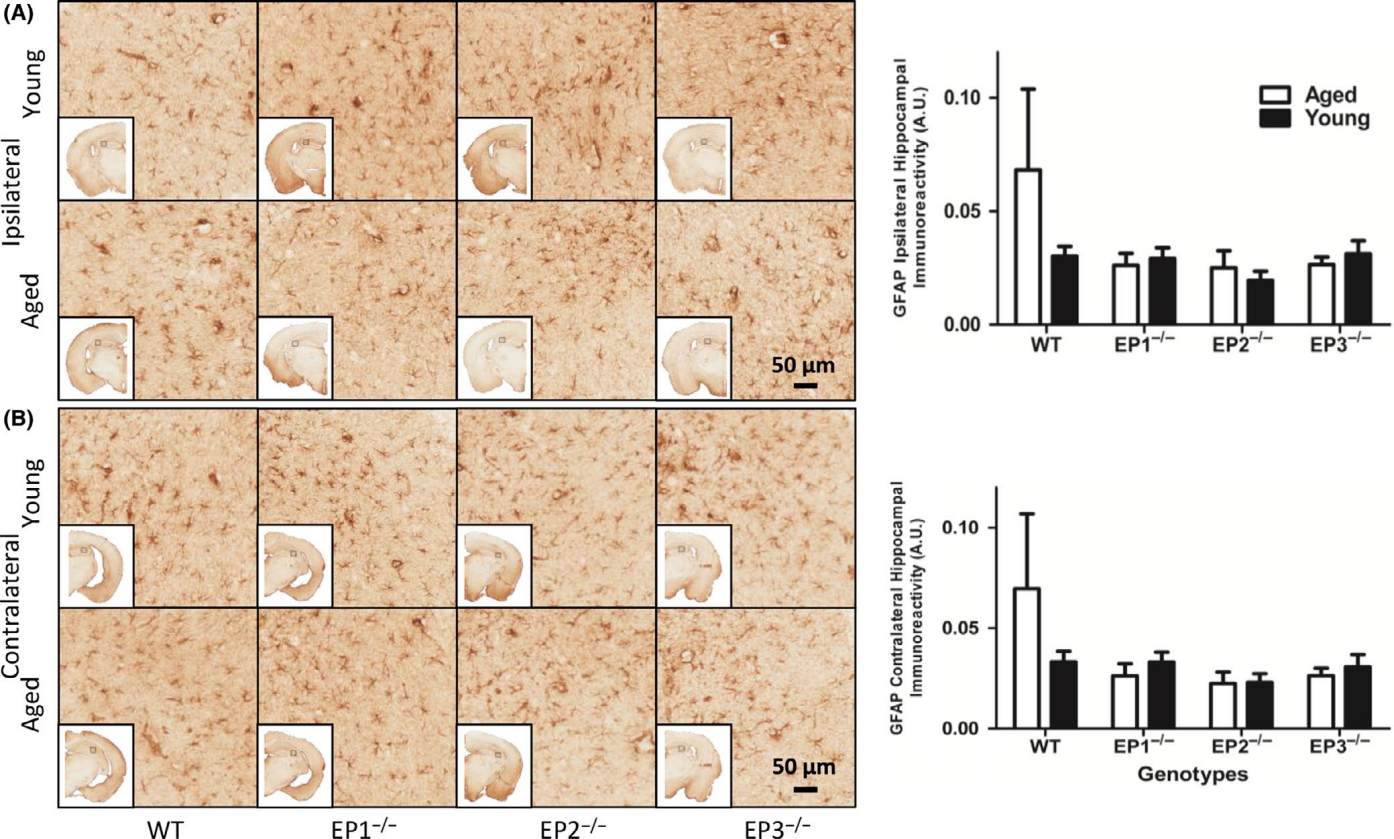
Effect of PGE_2_ EP1, EP2, and EP3 receptor deletion on astroglial activation after rCHI. Seventy‐two hours after TBI and WT, mice were sacrificed and brain sections were processed for GFAP immunochemistry to evaluate hippocampal astrogliosis and morphological changes. (A and B) Representative high magnification images of hippocampal brain sections showing the aged and young (A) ipsilateral and (B) contralateral for WT, EP1^−/−^, EP2^−/−^, and EP3^−/−^ mice. Square selections denote the magnified regions' locations. Quantification of brown positive pixel count demonstrated that no significant difference in GFAP immunoreactivity was found in (A) ipsilateral and (B) contralateral regions across both genotype and age groups. Comparisons included aged male and young male wild type (n = 8, n = 7), EP1^−/−^ (n = 10, n = 8), EP2^−/−^ (n = 8, n = 9), and EP3^−/−^ (n = 8, n = 11)

No statistical significance was found in hippocampal astrogliosis between any genotypes and age groups. Compared with WT aged controls, no statistical significance was found in the ipsilateral hemisphere of EP1^−/−^, EP2^−/−^, or EP3^−/−^ aged mice (0.068 ± 0.036 vs 0.026 ± 0.005 AU, *P* *=* .168, vs 0.025 ± 0.008 AU, *P* *=* .272, vs 0.026 ± 0.003 AU, *P* *=* .421; Figure 2A). Similarly, compared with WT aged controls, no difference was found in the contralateral hemisphere of EP1^−/−^, EP2^−/−^, and EP3^−/−^ aged mice (0.069 ± 0.037 vs 0.026 ± 0.006 AU, *P* *=* .143, vs 0.022 ± 0.006 AU, *P* *=* .164, vs 0.026 ± 0.004 AU, *P* *=* .513; Figure 2B).

In the ipsilateral hemisphere, no statistical significance was observed when EP1^−/−^, EP2^−/−^, and EP3^−/−^ young mice were compared with WT young controls (0.030 ± 0.004 vs 0.029 ± 0.005 AU, *P* = .877, vs 0.019 ± 0.004 AU, *P* *=* .098, vs 0.031 ± 0.006 AU, *P* *=* .915; Figure 2A). Similarly, no difference was observed in the contralateral hemisphere when EP1^−/−^, EP2^−/−^, and EP3^−/−^ young mice were compared with WT young controls (0.033 ± 0.005 vs 0.033 ± 0.005 AU, *P* = .994, vs 0.023 ± 0.004 AU, *P* *=* .161, vs 0.031 ± 0.006 AU, *P* *=* .793; Figure 2B).

No statistical significance in hippocampal astrogliosis was observed in the ipsilateral hemisphere when EP1^−/−^ aged mice were compared with EP2^−/−^ aged mice and EP3^−/−^ aged mice (0.026 ± 0.005 vs 0.025 ± 0.008 AU, *P* *=* .759, vs 0.026 ± 0.003 AU, *P* *=* .462; Figure 2A). When EP2^−/−^ aged mice were compared with EP3^−/−^ aged mice, similar results were observed (0.025 ± 0.008 vs 0.026 ± 0.003 AU, *P* *=* .865). No statistical significance was observed in hippocampal astrogliosis in the ipsilateral hemisphere when EP1^−/−^ young mice were compared with EP2^−/−^ young mice and EP3^−/−^ young mice (0.029 ± 0.004 vs 0.019 ± 0.004 AU, *P* *=* .150, vs 0.031 ± 0.006 AU, *P* *=* .692). When EP2^−/−^ young mice were compared with EP3^−/−^ young mice, similar results were observed (0.019 ± 0.004 vs 0.031 ± 0.006 AU, *P* *=* .113).

Analogously, no statistical significance was observed in hippocampal astrogliosis in the contralateral hemisphere when EP1^−/−^ aged mice were compared with EP2^−/−^ aged mice and EP3^−/−^ aged mice (0.026 ± 0.006 vs 0.022 ± 0.006 AU, *P* *=* .854, vs 0.026 ± 0.004 AU, *P* *=* .358; Figure 2B). When EP2^−/−^ aged mice were compared with EP3^−/−^ mice, similar results were observed (0.022 ± 0.006 vs 0.026 ± 0.004 AU, *P* *=* .584). No statistical significance was observed in hippocampal astrogliosis in the contralateral hemisphere when EP1^−/−^ young mice were compared with EP2^−/−^ young mice and EP3^−/−^ young mice (0.033 ± 0.005 vs 0.023 ± 0.004 AU, *P* *=* .144, vs 0.031 ± 0.006 AU, *P* *=* .813). When EP2^−/−^ young mice were compared with EP3^−/−^ young mice, similar results were observed (0.023 ± 0.004 vs 0.031 ± 0.006 AU, *P* *=* .526).

### 3.3 Effect of EP1, 2, 3 receptor deletion on the passive avoidance test in young and aged mice after rCHI

**Figure 3:**
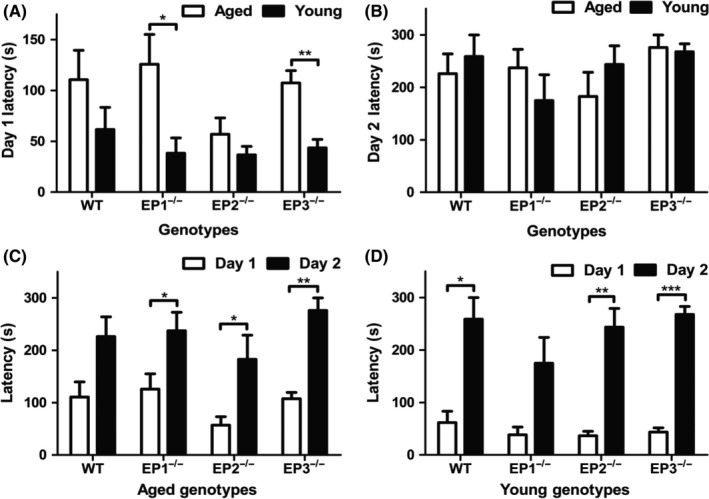
Effect of PGE_2_ EP1, EP2, and EP3 receptor deletion on passive avoidance memory test after rCHI. On day 1, mice were trained in passive avoidance apparatus and 24 hours later an identical test trial was performed in which the shock was omitted. (A) Both EP1^−/−^ and EP3^−/−^ aged mice had significantly greater latency time on day 1 than their respective young counterparts. (B) On day 2, no significant differences in latency time were observed between aged and young mice across the genotypes. (C) On day 2, significantly higher latency time was observed in EP1^−/−^, EP2^−/−^, and EP3^−/−^ aged mice compared with day 1. (D) For WT, EP2^−/−^, and EP3^−/−^ young mice, the latency time was significantly higher on day 2 compared with day 1. Comparisons included aged male and young male wild type (n = 8, n = 7), EP1^−/−^ (n = 10, n = 8), EP2^−/−^ (n = 8, n = 9), and EP3^−/−^ (n = 8, n = 11), *P < .05, **P < .01, ***P < .001

The latency of aged and young age groups of different genotypes was compared at various time points post‐RCHI. EP1^−/−^ young mice had a significantly lower latency than EP1^−/−^ aged mice on day 1 (38.2 ± 15.1 vs 174.8 ± 49.4 seconds, *P* *=* .021; Figure 3A). Similar observations were seen within the EP3^−/−^ genotype, in which young mice had significantly lower latency than aged mice on day 1 (43.5 ± 8.3 vs 107.5 ± 12.1 seconds, *P* *=* .003). On day 2, no significant differences in latency were observed between aged and young mice across the genotypes (Figure 3B).

There was a significant difference in latency time within EP1^−/−^ aged mice from day 1 to day 2 (125.9 ± 29.2 vs 237.3 ± 35.3 seconds, *P* *=* .035; Figure 3C). On day 2, the latency time was significantly greater for EP2^−/−^ aged mice than on day 1 (57.0 ± 15.9 vs 182.8 ± 45.9 seconds, *P* *=* .031). There was also a significant difference in latency time within EP3^−/−^ aged mice from day 1 to day 2 (107.5 ± 12.1 vs 275.9 ± 24.1 seconds, *P* *=* .002).

For WT young mice, there was a significant improvement in latency from day 1 to day 2 (61.7 ± 21.6 vs 258.8 ± 41.2 seconds, *P* *=* .017; Figure 3D). Similar to WT young mice, EP2^−/−^ and EP3^−/−^ young mice experienced a significant improvement in latency from day 1 to day 2 respectively (36.7 ± 8.3 vs 243.8 ± 35.6 seconds, *P* *=* .002; 43.5 ± 8.3 vs 267.9 ± 15.2 seconds, *P* < .0001). In further statistical analysis, the ratio of day 2 to day 1 latency was also compared between genotypes. EP3^−/−^ young mice had a significantly higher ratio than EP3^−/−^ aged mice (8.7 ± 1.7 vs 2.8 ± 0.5 seconds, *P* *=* .001).

## 4 DISCUSSION

In previous in vivo and in vitro studies, selective agonists/antagonists of different prostanoid receptors have been considered as a potential alternative treatment in place of COX inhibitors for neurological conditions such as ischemic stroke and TBI that involve acute excitotoxicity.^7^, ^12^, ^20^, ^21^, ^22^, ^23^ Various teams, including ours, have studied the effects of the different types of PGE_2_ receptors separately in different neurological conditions, but there are currently no studies to our knowledge that have compared the effects of these receptor subtypes in CHI. Neuroinflammation following a TBI involves both astrogliosis and microgliosis.^14^, ^15^, ^24^, ^25^, ^26^ Since neuroinflammation may have either neuroprotective or neurotoxic effects, astrogliosis and microgliosis may be either beneficial or harmful depending on their respective activation levels.^14^, ^15^, ^27^ The overactivation of astrocytes and microglia may result in undesirable secondary damage following TBI.^1^, ^7^, ^14^, ^15^ Since most clinical care of TBI patients focuses on the secondary damage associated with the primary brain trauma, mitigating astrogliosis and microgliosis is a significant clinical treatment objective.^1^, ^2^, ^28^, ^29^, ^30^ This study uniquely aims to quantify astrogliosis and microgliosis in PGE_2_ receptor knockout mice to start understanding which of the EP1, 2, and 3 receptor subtypes leads to the overactivation of astrocytes and microglia and may therefore represent potential targets for the clinical treatment of the secondary damage associated with brain trauma. We found that EP1 deletion in aged mice resulted in significantly lower microgliosis in both the contralateral and ipsilateral brain regions when compared to other genotypes and age groups. Among the aged mice, EP1 receptor deletion resulted in the highest reduction in microgliosis compared with EP2 and EP3 receptor deletion. EP1 receptor deletion was also found to be significantly more effective in reducing microgliosis in aged mice relative to the young mice. However, no statistically significant differences were discovered in astrogliosis between genotypes or age groups. With passive avoidance testing, we found that there was a significant increase in latency time for aged EP1^−/−^, EP2^−/−^, and EP3^−/−^ mice from day 1 to day 2. Similar observations were also noted in young mice in which WT, EP2^−/−^, and EP3^−/−^ mice experienced a significant increase in latency time from day 1 to day 2. When compared between age groups at different time points, EP1^−/−^ and EP3^−/−^ young mice had significantly lower latency time on day 1 when compared to their aged counterparts.

Through previous studies, it is well established that age influences microglia activation.^31^, ^32^ It has also been observed that the microglial response to experimental TBI was higher and prolonged in aged mice when compared to young mice. The aged mice (21‐24 months) also had increased microglia activation in the hippocampus compared with the young mice (5‐6 months).^31^, ^33^, ^34^, ^35^ The likely explanation for such observations is that microglia in the aged brain may be more primed to respond quickly and generate greater inflammatory responses relative to microglia in the younger brain.^31^, ^33^, ^34^, ^35^, ^36^ The excessive amount of microglial activation following TBI may lead to increased neuronal loss in the hippocampus and worse neurological outcome in the elderly.^37^, ^38^, ^39^, ^40^, ^41^, ^42^ In this study, we observed that the aged mice with EP1 deletion experienced a significantly lower amount of hippocampal microgliosis compared with aged WT mice. The EP1^−/−^ aged mice also experienced a significantly lower amount of hippocampal microgliosis than the EP1^−/−^ young mice. Relative to the aged EP2^−/−^ and EP3^−/−^ mice, EP1^−/−^ aged mice experienced a significantly lower amount of hippocampal microgliosis. This indicates that the activation of the EP1 receptor might be the primary route through which microglial activation occurs in aged mice in the setting of rCHI. Since the excessive microglial activation in aged mice has been shown to result in significant neuronal loss, our finding suggests a possible route that can be controlled through the application of antagonists or genetic deletion to decrease the microglia activation response. Our observations also indicate that the pathways for microglia activation may be age‐ and receptor‐dependent since similar results were not observed in the young mice. One possible explanation may be that some EP receptors are greatly upregulated in the microglia of aged mice or there are some compensatory mechanisms in the knockouts. The data from this preclinical model also have clinical relevance as the highest rates of TBI‐related deaths and hospitalizations have been observed in persons aged 75 years and older. Most of these cases were primarily related to falls and mild CHI.^5^ Further studies need to be conducted to elucidate how different pathways are over‐ or under‐utilized to activate microglia based on age. We found no significant difference in astrogliosis between either genotype or age group. This finding supports in part our previous finding that demonstrated neither the deletion nor activation of the EP1 receptor has a significant effect on astrogliosis in a controlled cortical impact (CCI) model.^20^ It suggested that astrocyte markers may not be directly affected by EP1 receptor activity and are probably linked with other components of injury, such as other prostaglandin receptors.^20^ Since there was no significant difference in astrogliosis in EP2^−/−^ and EP3^−/−^ mice when compared to WT mice, our findings indicate that the deletions of EP receptors do not affect astrogliosis under this relatively mild concussion model. Thus, collectively these studies suggest astrocytes may be linked with other mechanisms of injury that may not involve EP receptors.

A passive avoidance test was employed because it is an appropriate behavioral paradigm to assess short‐ and long‐term memory. It is also suitable for initial screening, as it is relatively easy to set up and perform and is sensitive to both rat and mouse TBI models.^43^ Upon passive avoidance testing, we discovered that EP1^−/−^, EP2^−/−^, and EP3^−/−^ aged mice experienced a significant increase in latency time from day 1 to day 2. This suggests that even though this repetitive CHI resulted in some cognitive deficits on day 1 in the mice of all genotypes, the deletion of EP receptors resulted in faster recovery than WT mice. EP1^−/−^ young mice experienced no significant difference in latency from day 1 to day 2. Similar to anatomical changes, the behavioral findings show that EP1 receptor activation plays a more prominent role in determining outcomes following rCHI in aged mice. It indicates that the activation of the EP receptors, specifically the EP1 receptor, may play a role in the delayed improvement in the cognitive deficits after TBI in the aged mice. Since the ability of the mice to learn a task did not diminish after mild TBI, our finding further confirms the behavioral outcomes of previous studies that used CHI mouse models.^20^, ^35^, ^43^, ^44^ In general, both young and aged mice across different genotypes did not experience cognitive deficits on day 2. Therefore, this mild repetitive concussion paradigm may lead to temporary cognitive deficits, but overall improvements in cognition over time.

Further studies need to be conducted to confirm and extend these findings and should focus on understanding the mechanisms of the EP receptors to gain additional information on how different receptor modulators can be utilized in acute brain injuries. Some of our findings would appear to be different from previous studies due to the differences in the experimental paradigm. Quantification and time points utilized could also contribute to some differences.

In conclusion, the data from this study extend our previous understanding of the role of the EP1, 2, and 3 receptors in TBI. It demonstrates how the neuroprotective and neurotoxic pathways that are involved after brain trauma might be dependent on the temporal modulation of the EP receptor‐mediated neuroinflammatory pathways. More detailed studies need to be conducted to further study the temporal profiles of these different EP receptors and their role in concussions/TBI. Our study suggests that the targeted application of different EP receptor modulators can serve as a potential treatment of TBI and other acute brain injuries.

## CONFLICT OF INTEREST

The authors declare no conflict of interests.
